# Letter to the Editor: Surgical approach, preoperative LEEP/conization, and patterns of recurrence and death in low-risk cervical cancer – exploratory analysis from the CCTG CX.5/SHAPE trial

**DOI:** 10.1097/JS9.0000000000003205

**Published:** 2025-08-07

**Authors:** Yanan Duan, Aiping Chen

**Affiliations:** a Department of Gynecology, The Affiliated Hospital of Qingdao University, Qingdao, Shandong Province, China; b Qingdao Medical College of Qingdao University, Qingdao, Shandong Province, China

*Dear Editor,*

We read with great interest the exploratory analysis by Mahner *et al*, recently published in the *International Journal of Surgery*[1]. The authors assessed patterns of recurrence and death in low-risk cervical cancer patients undergoing simple or radical hysterectomy, emphasizing the potential protective effect of preoperative conization with negative surgical margins (R0) before hysterectomy. However, several aspects need more discussion. The letter to the editor is compliant with the TITAN Guidelines 2025—governing declaration and use of AI[2].

First, a key question raised by the analysis is whether the favorable outcomes observed in patients achieving R0 margins at preoperative conization reflect genuine procedural efficacy or simply identify a subgroup of patients with inherently favorable tumor biology. The authors imply that R0 status prevents intraoperative tumor dissemination during minimally invasive surgery. However, it is suggested that tumors amenable to complete resection via conization are often smaller, exhibit less aggressive histological features, and possess intrinsically lower metastatic potential[3]. Without centralized pathological review or molecular characterization of tumor specimens, the analysis presented in the SHAPE study cannot distinguish whether the favorable outcomes result from surgical intervention or merely represent a surrogate marker of low-risk tumor behavior. Future studies should incorporate systematic central pathology reviews, detailed molecular profiling, and assessment of residual microscopic disease, such as circulating tumor DNA, to differentiate between these possibilities conclusively[4].

Secondly, the authors compare minimally invasive surgery and open surgery without clearly distinguishing among various techniques within the minimally invasive category. It is essential to recognize that minimally invasive surgery is not a single uniform approach. Surgical nuances, such as the use of uterine manipulators, methods of vaginal colpotomy, pneumoperitoneum pressure, and robotic versus conventional laparoscopy, have individually been shown to affect cancer recurrence rates significantly[5]. The SHAPE trial did not report such technical details, leaving open the possibility that combining different minimally invasive techniques into one analysis might obscure specific risk factors. We recommend future investigations systematically document procedural variations and conduct subgroup analyses based on surgical details to identify the truly safe minimally invasive surgical methods suitable for routine clinical practice.

Thirdly, while the SHAPE trial focuses primarily on oncologic outcomes, preoperative procedures such as loop electrosurgical excision procedure or conization can generate significant psychological distress, including anxiety about incomplete treatment or recurrence[6]. These factors may influence surgical decision-making, adherence, and recovery. Incorporating structured preoperative counseling and psychological support into surgical planning may improve patient preparedness and satisfaction.

In conclusion, Mahner *et al* provide valuable data on preoperative conization and minimally invasive surgery in low-risk cervical cancer. However, further clarification is needed before broad clinical adoption. Specifically, distinguishing whether R0 margins reflect therapeutic effect or tumor biology, disaggregating minimally invasive techniques, and addressing psychological distress associated with preoperative procedures are essential.

## Data Availability

All data are included in this article.
